# Movement-Based Estimation and Visualization of Space Use in 3D for Wildlife Ecology and Conservation

**DOI:** 10.1371/journal.pone.0101205

**Published:** 2014-07-02

**Authors:** Jeff A. Tracey, James Sheppard, Jun Zhu, Fuwen Wei, Ronald R. Swaisgood, Robert N. Fisher

**Affiliations:** 1 U.S. Geological Survey, Western Ecological Research Center, San Diego Field Station, San Diego, California, United States of America; 2 San Diego Zoo Institute for Conservation Research, Escondido, California, United States of America; 3 Department of Statistics and Department of Entomology, University of Wisconsin – Madison, Madison, Wisconsin, United States of America; 4 Key Laboratory of Animal Ecology and Conservation Biology, Institute of Zoology, Chinese Academy of Science, Beijing, People's Republic of China; Institut Pluridisciplinaire Hubert Curien, France

## Abstract

Advances in digital biotelemetry technologies are enabling the collection of bigger and more accurate data on the movements of free-ranging wildlife in space and time. Although many biotelemetry devices record 3D location data with *x*, *y*, and *z* coordinates from tracked animals, the third *z* coordinate is typically not integrated into studies of animal spatial use. Disregarding the vertical component may seriously limit understanding of animal habitat use and niche separation. We present novel movement-based kernel density estimators and computer visualization tools for generating and exploring 3D home ranges based on location data. We use case studies of three wildlife species – giant panda, dugong, and California condor – to demonstrate the ecological insights and conservation management benefits provided by 3D home range estimation and visualization for terrestrial, aquatic, and avian wildlife research.

## Introduction

Biologists have sought to understand the patterns of space use of individual animals for decades.

Burt [1] provided a basic definition of a home range as “that area traversed by an individual in its normal activities of food gathering, mating, and caring for young.” His widely accepted description became a conceptual underpinning for the development of empirical home range estimators used to model animal spatial behaviors and, more recently, an animal's cognitive map of its environment [2], [3]. Furthermore, home range size is related to vulnerability to extinction [4], [5]. The increasing sophistication and miniaturization of biotelemetry devices (biologgers) with global positioning system (GPS) capability are enabling data of unprecedented detail to be collected on the spatial behaviors of a widening diversity of animals. Home range estimators continue to be refined to capitalize on the increasing size, accuracy, and deployment lengths of animal tracking data sets, bringing inferences on animal space use closer to biological reality and to Burt's original definition of a home range (see reviews by [6]–[9]). These advances are enhancing our understanding of animal spatial behaviors and ecology, including resource use, dispersal, and population dynamics [2]. However, progress has been limited by the inability of existing modeling techniques to take advantage of the three-dimensional (3D) data sets, constraining estimates of animal space use to an often biologically unrealistic 2D “Flatland” [10]. Here, we present the next evolution of home range estimators for analyzing biotelemetry data and visualizing animal space use in 3D and demonstrate their considerable advantages over traditional 2D approaches.

**Figure 1:**
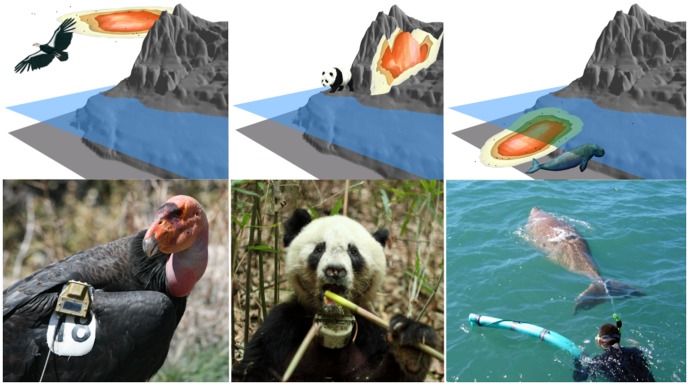
Example avian, terrestrial, and aquatic animal biotelemetry data sets and their spatial domains. Left: California condor with a GPS biologger attached to its patagium. Center: A giant panda telemetered with a GPS collar. Right: A dugong fitted with a tail mounted GPS biologger.

Animal space use can be characterized by the (*x, y*) spatial dimensions as well as a third *z*-dimension representing altitude, elevation, or depth for flying, terrestrial, or aquatic species, respectively ([11]; Figure 1). There are currently more than a dozen manufacturers offering wildlife biologgers that record 3D location data. Remote sensing technologies, such as light detection and ranging (LiDAR) sensors, can also be used to acquire 3D representations of animal habitats [12]–[14]. Yet, analyses of animal space and habitat use have typically been 2D, with the *z*-dimension examined separately or simply ignored [15]–[17]. Disregarding the *z*-dimension limits our understanding of spatial behaviors in relation to environmental heterogeneity [11] and may misrepresent the space use of animals that occupy habitats with a strong vertical component, such as mountains or undersea canyons [18]. Biologists are only beginning to recognize the theoretical and applied value of incorporating the vertical aspect into analyses of animal space use [18], [19].

We present a novel 3D movement-based kernel density estimator (MKDE) of animal home ranges and demonstrate the application and value of these estimators using biotelemetry data acquired from endangered animals that occupy aerial, terrestrial, and aquatic spatial domains. We also present a novel MKDE-based approach for calculating the spatio-temporal interaction between two individuals. We show that analyses and visualization using 3D MKDEs can be more informative and yield greater ecological insights than traditional 2D estimators in representing the space use of animals that have a substantive vertical component (Figure 1).

## Methods

A utilization distribution (UD) describes the probability of an animal location at an arbitrary time during which the animal was observed [20]. The kernel density estimator (KDE; [21]), which uses a weighted sum of kernels placed over observed animal locations [22], has become a standard technique for estimating home ranges. More recently, a Brownian bridge movement-based approach [23] provides an alternative KDE that integrates kernels over time along a movement path interpolated between observed locations. Benhamou [24] distinguished Worton's location-based kernel density estimators (LKDE) from movement-based kernel density estimators (MKDE), which includes Brownian bridge and biased random walk models [23], [24].

LKDEs are criticized for excluding areas that have been used by animals with large data sets (type I errors, [25]) while including areas that have not been used due to over-smoothing with small data sets (type II errors). Biotelemetry data sets with relatively short time intervals between locations often need to be subsampled to induce independence. In contrast, MKDEs account for time between consecutively observations in the estimator, do not require independent samples from the UD, and thus more realistically represent the space used by an animal. Therefore, our development of 3D methods is movement based [23].

### Biotelemetry Data

We use animal location data in which each observation includes an *x*-coordinate, a *y*-coordinate, a *z*-coordinate, and time. Let *m = 0, …, n* index observations (*x_m_, y_m_, z_m_*, *t_m_*) where *x_m_*, *y_m_*, and *z_m_* are the spatial coordinates and *t_m_* is the time of the observation such that *t_m-1_ < t_m_ < t_m+1_* for any *m*. Further, we assume that the observed locations are subject to observation error and are normal random variables described by 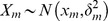, 
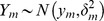
, and 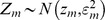. The observation error variances 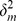 and 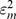 are either provided by the manufacturers of the telemetry equipment or estimated from field trials. The *m^th^* move step is defined by two consecutive observations *m* and *m+1*. We only use move steps that satisfy the conditions that 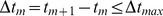 and 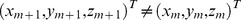. The first condition ensures that the time interval between locations is consistent with the study design and the second condition ensures that it is probable that the animal moved between observations. We check the second condition by assuming that 
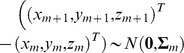
 where 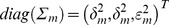 and all off-diagonal elements are 0. If 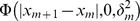, 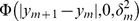, or 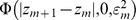
> 0.975, where 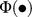 is the normal cumulative distribution function, we conclude that the animal moved during the time interval *t_m_* to *t_m+1_*. In other words, if the displacement in any spatial dimension is sufficiently improbable based on a random draw from the observation error distribution, we conclude that the animal actually moved. For 3D analyses, we impose the additional requirement that *z_m_* is within the range the animal is allowed to occupy. For birds as an example, *z_m_* must usually be at or above ground level, but in some cases this may not occur if the GPS receiver fails to obtain a 3D fix. We use *I(m)* as an indicator function that yields a value of 1 when the *m^th^* move step meets these conditions and 0 otherwise.

### 2.5D Movement-Based Kernel Density Estimator

Projecting a terrestrial animal's UD onto a 2D plane systematically underestimates the area used if the terrain is not a level, flat surface. As the curvature of the terrain increases, the underestimation becomes more severe. Thus, we demonstrate a 2.5D approach for computing home range area that essentially uses a 2D MKDE draped over a 2D elevation raster [26]. We correct the bias by calculating and summing the surface area of each cell of the elevation raster that falls within a desired probability contour of the 2D MKDE. We used the tessellation algorithm developed by Jenness [26] to compute the surface area of each raster cell. This method uses the cell center coordinates and elevations of the focal cell and its eight neighboring cells to construct eight triangular facets within the focal cell. We modified Jenness's approach to use bilinear interpolation to compute the elevation at the eight vertices (at the four corners and midpoint of each side) of each focal cell. The area of each facet is calculated using Heron's formula and then summed to obtain the surface area for the focal cell. Finally, we incorporate the area calculation into the probability of use of each cell by multiplying the probability density at the center of the cell by the computed cell surface area, and then normalize by dividing this product in each cell by the sum of the density-area products over all cells.

### 3D Movement-Based Kernel Density Estimator

#### 3D Kernel

We estimate the 3D MKDE using a trivariate normal kernel integrated over time for each observed move step. The kernel describing the probability density at time *t* and location (*x,y,z*) is
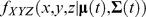



where the vector of means is 
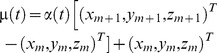
 = 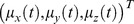. Thus, we approximate the true movement path by linearly interpolating between consecutive observed locations. The covariance matrix is assumed to be
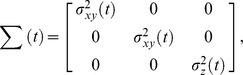
where the time-dependent variance in the *x*-dimension and *y*-dimension is 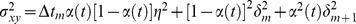 and the time-dependent variance in the *z*-dimension is 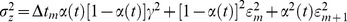. The length of the time interval associated with the time step is 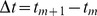, the proportion of the time interval between *t_m_* and *t_m+1_* at time *t* is 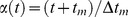, and *t* implies *m* by 
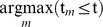
. Notice that the contribution of the move variance parameters 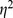 and 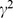 to the time-dependent kernel variance is small when *t* is close to *t_m_* or *t_m+1_*, and increases as time is further away from the observation times. These move variance parameters are diffusion coefficients [27] expressed in units of (distance^2^)/time. Thus, when preparing the data, we express time in smaller time units such as minutes, which reduces the magnitude of the parameter and helps avoid numerical issues. The move variance parameters *η^2^* and *γ^2^* are estimated from the data using a likelihood-based approach by numerically maximizing the log-likelihood function using alternating observed locations as described by Horne et al. [23].

Because we assume that the off-diagonal elements of the covariance matrix are zero and that the variances in the *x*-dimension and *y*-dimension are equal, our kernel is the product of univariate distributions in each dimension given by
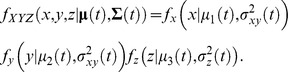
This assumption can be exploited to increase the computational efficiency of the 2D or 3D MKDE calculations.

#### Utilization Distribution

The density for the Brownian bridge for the *m^th^* move step is obtained by integrating a normal kernel over time: 


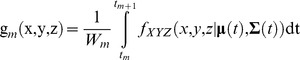
 [23]. We normalize the density for the single move step by dividing the integral by
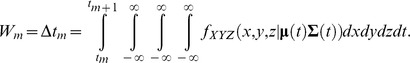
To compute the full UD, we sum over all move steps and normalize the result by
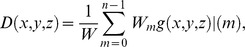
where the normalization constant is 
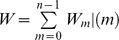
. However, if we restrict the space available for use by an animal, we must take this reduced domain into account when normalizing the utilization distribution.

#### Bounding in the z-dimension

Often, an animal's movement is limited in the *z*-dimension. For example, avian species are generally bounded below by the earth's surface, whereas marine animals are bounded below by the sea floor and above by the water's surface. We can bound the density in the z-dimension by *a(x,y)* and *b(x,y)* with a constant, a 2D raster, or a function. However, if we bound the density in the z-dimension by *a(x,y)* and *b(x,y)*, the limits of integration in the z-dimension must be modified when computing the normalization constant for each move step:
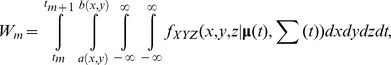
Applying a kernel density estimator to a bounded region, as noted by Silverman [21], tends to produce an underestimate of the density near the boundaries. This is because there are no points across the boundary outside the interval [*a(x,y), b(x,y)*] to contribute to the density estimate. One common approach to reduce the bias is to augment the data with additional observations by reflecting the data about the boundaries [21]. In the MKDE approach, this amounts to reflecting the interpolated move path for each move step about the boundaries. Since we are bounding space in the *z*-dimension, we augment the data with 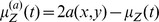 and 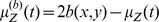. In practice, we need only reflect the move paths about the boundary 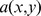 when 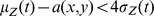 and about 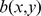 when 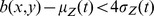 [21]. We apply this technique in our 3D MKDE approach.

#### Probability Estimation on a Regular Grid

An MKDE can be integrated to compute the probability for any area in 2D space or volume in 3D space, but to visualize the 3D MKDEs across space we compute the probability for every voxel (3D cell) on a regular grid in 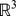 or every cell on a regular grid in 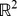. It is important that the 3D regular grid covers the non-negligible parts of the UD. Thus, we set the minimum size of the grid based on the range of the observed locations buffered by 4.265 standard deviations (or some other quantile of the standard normal distribution), where the standard deviation is determined by the maximum kernel variance 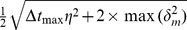 in the (*x*,*y*)-dimensions and 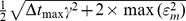 in the *z*-dimension.

We index rows as *i = 0,…,I-1*, columns as *j = 0,…,J-1*, and levels as *k = 0,…,K-1* where *I*, *J*, and *K* are the number of rows, columns, and levels, respectively. Rows, columns, and levels correspond to the *y*-, *x*-, and *z*-dimensions, respectively. Half the lengths of the sides of a voxel in each dimension are *h_x_*, *h_y_*, and *h_z_*. For simplicity, we index voxels by *v = i+j×I+k×I×J*. Let the random variable V be the voxel in which the individual is found at some random time during the move steps used to compute the 3D MKDE.

The probability of an individual being in voxel *v* during move step *m* as a function of time *t* is 
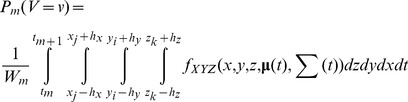
and the probability that an animal will be in voxel *v* over all move steps is
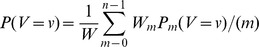
We can similarly compute the probabilities for cells in a 2D MKDE. It is this probability that we compute in the case studies that follow. We interpret these probabilities as the proportions of time spent in each voxel (or cell for the 2D case) during the time intervals between observed locations used to compute the MKDE.

In practice, when we set a lower or upper bound on the 3D MKDE in the *z*-dimension, we use either a constant or a 2D raster with the same origin, number of rows, number of columns, number of levels, and cell or voxel sizes as the MKDE. Furthermore, we assume that *a*(*x,y*) and *b*(*x,y*) fall on the boundaries of the voxels in the *z*-dimension so that each voxel is either entirely within or outside the boundary. As a result, *a*(*x,y*) and *b*(*x,y*) are constant within a given voxel.

#### Spatio-temporal Interaction between Two Individuals

Describing the interaction between two individuals is often of interest to ecologists because it relates to social interaction, transmission of infectious disease, and other individual-level ecological processes. Various approaches have been developed for describing interactions between individuals based on movement data and UDs in 2D space [28], [29]. Here we present a novel approach that uses the temporally-explicit nature of 2D and 3D MKDEs to quantify the interaction between two individuals, labeled A and B, in both space and time. Let *V_A_* and *V_B_* be random variables for the voxel in which animal A and B, respectively, are found at some given time *t*. Following Bhattacharyya's coefficient [30], we compute the square root of the product of the kernels for both animals at an arbitrary location (*x,y,z*) and time *t* during move step *m* by
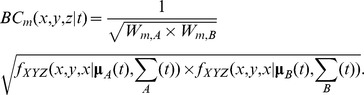
We can then, as described above for a single individual, integrate this function over the area of each cell or volume of each voxel and time within move steps and sum across move steps to obtain a local (cell or voxel) measure of spatial-temporal similarity between the individuals that increases at the increase of similarity between the kernels of the two animals over the time interval of each move step. When we sum this value over all cells or voxels, we obtain a global measure of spatio-temporal similarity that ranges from 0 (no similarity) to 1 (perfect spatio-temporal overlap).

#### Computational Considerations

All calculations are performed using code written by the authors in R and C++, and use the raster, Rcpp, and ggplot2 packages in R [31]–[33]. When computing the probability of an individual occurring in a given voxel, we exploit the independence assumptions that allow us to express the join density in 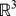 as the product of the univariate densities in each spatial dimension. Output is written as tabular data in CSV format as ASCII VTK (Visualization Toolkit, [34]) files. Results are visualized using ParaView [35] and the R package ggplot2.

We determined the voxel or cell probabilities corresponding to contours delineating the minimum volume or area containing a user-specified proportion (e.g., 0.99) of the total UD as follows. First, vectorize the 3D or 2D array containing the voxel or cell probabilities (so that it becomes a 1D array). Second, sort the resulting 1D array in ascending order using a fast sorting algorithm such as quicksort. Third, create a 1D array of the same length containing a cumulative sum of the sorted values in step 2. Fourth, find the index of the entry in the array created in step 3 that most closely matches one minus the user-specified proportion. Fifth, return the voxel or cell probability at that index in the sorted array created in step 2. Every voxel or cell with a probability greater-than-or-equal to this value is included within the contour and excluded otherwise.

### Terrestrial Example: Giant Panda

We applied 2.5D MKDE to data for a free-ranging giant panda (*Ailuropoda melanoleuca*). The giant panda is listed as an endangered species by the International Union for Conservation of Nature (IUCN) and a Class 1 Protected Animal by the Chinese Government [36]. The panda population has decreased to < 2,500 individuals due to anthropogenic degradation and loss of bamboo habitat [36]. In a biotelemetry study in the Qinling Mountains of southwest China (33° 32′ – 33° 45′ N, 107° 40 – 107° 55′ E), veterinarians and field biologists at the Chinese Academy of Science tracked the pandas in the wild during the winter months between November and March, darted them using a compressed air gun delivering approximately 5 mg ketamine/kg body weight, and then fitted the pandas with a GPS collar. The GPS collars also recorded the animal's elevation above sea level (Lotek, GPS 4400M; [37]). Giant panda research methods were approved by the San Diego Zoo IACUC animal welfare committee (Project ID# 221), the National Natural Science Foundation of China (30830020, 30970392), and the Chinese Academy of Sciences (KSCX2-EW-Z-4). In this example, we used data from a 3.5 year-old adult male panda that was tracked for 473 days between February 2007 and January 2009. A total of 1,916 GPS locations were collected. We used a published equation for mean error for the same model of GPS telemetry unit as a function of percent forest canopy cover [38], assuming 50 percent cover, and the relation 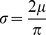 for a Rayleigh distribution to obtain an approximate measurement error variance (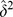) of 158.96 m^2^. Terrain surface area was computed using a 32 × 25 km, 30-meter resolution digital elevation model (DEM) with elevation ranging from 875 to 3,035 meters in the *z*-dimension [39], [40].

We compared home range areas estimated by the 2D MKDE versus our 2.5D MKDE that incorporates this rugged topography. We performed this comparison using all panda locations, locations in the summer at higher elevation, and locations in the winter at lower elevation. We allowed a maximum time between locations (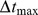) of 190 minutes and used a 0.5 minute time step for numerical integration. The 2D MKDE had the same 30 m resolution as the DEM.

### Avian Examples: California Condor

We applied the 3D MKDE to study free-ranging California condors (*Gymnogyps californianus*). Populations of this large vulture declined precipitously due to intentional shooting, lead poisoning from ingestion of spent ammunition in carcasses, egg collecting, and poisoning [41], [42]. Following an intensive captive breeding program, San Diego Zoo Global and its partner organizations successfully reintroduced condors to their former range in the Sierra San Pedro de Martir ranges of Northern Baja California, Mexico (Lat  =  31° 2′ 16.29″ N; Long  =  115° 35′ 1.56″ W). Before each condor was transported to Mexico for release, it was fitted with a patagial-mounted 50 g solar Argos PTT/GPS-transmitter (PTT-100, Microwave Telemetry Inc.) and ID tag in a captive environment by San Diego Zoo veterinarian staff [43]. Using manufacturer-provided data [44], we fit a normal distribution, taking both tails into account, to obtain a measurement error variance of 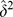  =  79.39 m^2^ and a Rayleigh distribution to obtain a vertical measurement error variance of 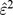  = 308.00 m^2^. The units are programmed with an hourly fix rate between 6:00am and 8:00pm and transmit location data via the Argos network. Research on California condors was approved by the United States Fish and Wildlife Service, the San Diego Zoo IACUC animal welfare committee (Project ID#11-014) and the Instituto Nacional de Ecología, México.

We present several examples using data from California condors reintroduced to Baja, Mexico. In all examples, a 2.5-minute integration time step was used. The avian MKDE was bounded below by elevation based on a DEM raster [39] that was 280 km by 445 km in the (*x, y*) dimensions and ranged from 0 m to 3,065 meters in the *z*-dimension with a cell resolution of 27.07 meters [40]. However, we aggregated these cells (by averaging) to coarser resolutions depending on the spatial extent covered by each MKDE. We used a maximum time between locations (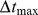) of 70 minutes.

First, we illustrate the process of constructing a 3D MKDE. In this example, we used 2,760 GPS location fixes from a 5-year-old adult female California condor collected over 214 days from December 2009 to July 2010 to compute a 3D MKDE at a 216.55 meter resolution. In a second example, we compared 3D MKDEs for an adult female and adult male condor that formed a breeding pair following reintroduction. These condors were tracked during 10 January – 9 March 2011, with locations being collected at the same times at one-hour intervals. In all, 1,132 temporally-matched locations were available for analysis. We used these data to compute 3D MKDEs at 108.28 meter resolution for each individual separately and also the probability of both members of the pair occurring in the same voxel at the same time using the spatio-temporal interaction 3D MKDE. We also use the data from the female of this pair to compare results from the 2D MKDE and 3D MKDE approaches. We compare contours of the 3D MKDE projected onto the 2D plane to contours of a corresponding 2D MKDE. Next, motivated by a 2D MKDE approach by Lewis et al. [45], we show how the 3D MKDE approach can be used to evaluate risk to wildlife using a proposed wind farm in Baja, Mexico as a case study. In April 2007, we tracked a subadult female condor making a directed long-distance flight 230 km from the reintroduction site in Baja California, Mexico north towards Southern California, USA. When this bird was 5 km south of the USA/Mexico international border we recorded two consecutive hourly fixes from its GPS transmitter that were spaced 16 km apart with a path step, that if interpolated linearly, traversed the planned Phase-1 turbine locations of the 156 MW Energía Sierra Juárez Wind Energy Project being developed by IENova/Sempra U.S. Gas and Power [46]. This linear step also spatially coincided with areas of the Sierra ranges previously modeled to have the highest consistent mean wind speeds in the Baja border region of 8.5–9.0 meters/second at heights ∼80 meters [47]. The project proposes the installation of Vestas V112-3.3 wind turbines with a tower height of 84 meters and a rotor radius of 56 meters (total height of approximately 140 meters). We computed 2D and 3D MKDEs for the single move step through using 54.14 meter cells and 54.14 meter voxels, respectively. At each turbine location, we extracted the 2D MKDE probability for the cell in which the base of the tower falls and summed the probabilities of the three voxels (162.42 meters) above ground level to estimate the probability that the condor would have collided with each turbine. Finally, we illustrated how to relate 3D MKDEs to 3D covariate data. We used 433 observations from an adult female condor collected during November, 2009 to develop a 3D MKDE. We matched the MKDE voxels to predicted mean wind speed for November 2009 generated using a custom wind speed climate model by Regional Earth System Predictability Research Inc. [48]. The RESPR model used proprietary atmospheric simulation technology and meteorology data sets produced by the National Centers for Environmental Prediction North American Model as input to a high-resolution (1-km) simulation of wind speed at the condor reintroduction site for November 2009. Wind speed was modeled from 14–150 m above the ground. Over the study area, predicted mean wind speeds ranged from 1.835 to 6.38 meters/second. We interpolated these predictions on a regular grid with voxel dimensions of 250×250×10 meters in x, y, and z, respectively.

### Aquatic Example: Dugong

We applied our 3D MKDE to study a free-ranging dugong (*Dugong dugon*), which are marine mammals in the order Sirenia, along with manatees. Dugongs inhabit an aquatic environment and are seagrass community specialists, and are listed by the IUCN as being vulnerable to extinction. Dugongs, like manatees, face numerous anthropogenic threats, including boat-strike, incidental capture in fishing nets, and habitat degradation [49]–[51]. In a study in Hervey Bay, Queensland, Australia (25° 11.4′ S, 152° 36.6′ E), wild dugongs ≥2 m long without an attendant calf were captured during winter (June – July) using the method of [52] and [53]. Total time to capture, tag, and release a dugong was typically 10–12 minutes. When operating in deep (>2 m) or turbid water, we used a spotter plane to locate dugongs. We also used the spotter plane to monitor the behavior and well-being of captured dugongs for 15–20 minutes post-release. Research on dugongs was approved by the James Cook University Animal Ethics Sub-Committee (Ethics Approval No.: A56900), the Permitting Committee of the Department of Environment and Water Resources under Section 266A of the Environment Protection and Biodiversity Conservation Act 1999, the Queensland Environmental Protection Agency (permits W4/002726/02/SAA and MP2002/005), and the Great Barrier Reef Marine Park Authority (permit G01/304). In this example, we used data for a 3 m long adult male dugong that was captured and tracked in Hervey Bay in July 2004. The dugong was fitted with an Argos PTT/GPS satellite tag (Telonics Inc.) and tracked for 41 days within its 23.8 km^2^ core seagrass habitat [53], [54]. The GPS tag had a 20-minute fix cycle every 24 hours and provided 839 GPS locations with a fix success rate of 85%. Fitting a Rayleigh distribution to published data for the same model of GPS unit (Deutsch, Edwards, and Barlas. 2006) we estimated a measurement error variance in the (*x, y*)-dimension (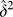) of approximately 25.0 m^2^. An Mk9 timed-depth recorder (TDR, Wildlife Computers) was fitted to the satellite tag harness at the base of the dugong's tail to record its dive profile (*z*-dimension). The TDR measures the depth of the dugong's tailstock, which can be at a maximum possible difference of approximately 2 m from the dugong's head depending on its orientation [55], although this maximum distance is typically observed only when dugongs are swimming at higher speeds (JS, pers. obs). Therefore, this potential variability is likely negligible if the dugong is in very shallow <2 m or deep water >5 m, but may introduce noise if the animal is in moderately shallow water 2–5 m. The TDR had a 25 cm depth resolution (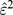 = 0.0625 m^2^) and an accuracy of ±1% and a continuous 1-second sampling interval. The dugong MKDE was bounded below by a 4.3×6.8 km, 10 m resolution bathymetric surface of the core seagrass habitat in depth below the mean sea level, which ranged from 6.1 m to -1.2 m [56]. We used voxel dimensions of 10×10×0.5 meters (x, y, and z, respectively) to compute the 3D MKDE probabilities.

To examine dugong space use patterns in relation to tidal height, the data set was divided into locations that occurred in five tidal height ranges of 0.5–1.0 m, 1.0–1.5 m, 1.5–2.0 m, 2.0–2.5 m, and 2.5–3.0 m. For each tidal height range, the dugong 3D MKDE was bounded below by the raster describing bathymetry and above by a constant water level above low tide based on the upper limit of the tidal height range. Thus, in this example, we accounted for the temporally dynamic space that dugongs inhabit. We allowed a maximum time between locations (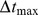) of 25 minutes and computed the 3D MKDE using a 1-minute time step for numerical integration. The 3D MKDE raster had a resolution of 10 m in the (*x, y*)-dimensions (the same resolution as the bathymetry raster) and 0.5 m in the *z*-dimension. Using the 3D MKDEs for each tidal range, we estimated the probability that the dugong would have occurred at various depths, and related these probabilities to dugong availability 1.5-meter and 2.5-meter detection zones often used for aerial surveys [57].

## Results

### Giant Panda

When using all locations, 650 of the 1,916 total observed locations satisfied the conditions for use in the 2D MKDE and 678 were removed because we concluded that the following location did not represent a large enough displacement to be considered a move step. Based on the 650 move steps, we estimated 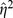  = 328.59 m^2^/min. For locations in the winter range, 167 of the 479 total observed locations satisfied the conditions for use in the 2D MKDE and 177 were removed. Based on the 167 move steps, we estimated 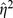  = 34.51 m^2^/min. For locations in the summer range, 479 of the 1425 total observed locations satisfied the conditions for use in the 2D MKDE and 500 were removed. Based on the 479 move steps, we estimated 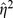  = 425.80 m^2^/min. Twelve locations were not used because they represented migratory movements between the winter and summer ranges.

**Figure 2:**
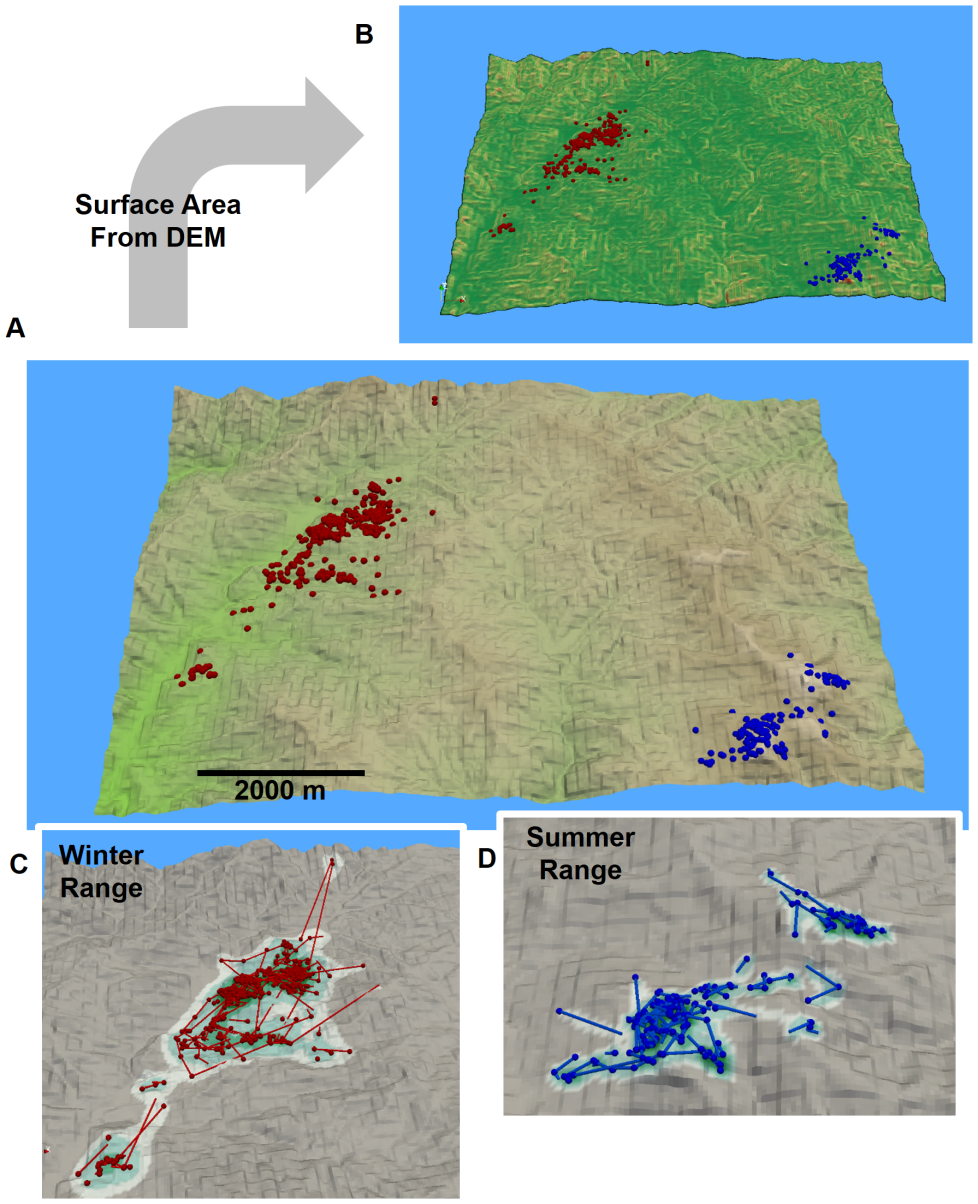
An example of a 2.5D MKDE for a giant panda in rugged terrain. In A, giant panda GPS locations in its summer (red points) and winter (blue points) ranges are shown in relation to a digital elevation model (DEM). Using the DEM, the surface area of each raster cell is calculated (B). The surface area increases as the color gradient changes from green to red. In C, the observed summer range locations and interpolated move paths (red points and lines) are shown against 2D MKDE contours draped over the DEM. In D, the observed winter range locations and interpolated move paths (blue points and lines) are shown against 2D MKDE contours draped over the DEM. 2D MKDE 99%, 95%, 75%, 50% contours are shown with colors ranging from light to dark green.

Figure 2 illustrates the giant panda 2.5D MKDE and cell surface areas. Surface areas based on 2.5D MKDE ranged from 900.0 to 1998.6 m^2^ (mean  = 1024.9 m^2^, sd  = 158.8 m^2^) and showed a 12.09% to 16.82% relative increase over the estimates based on 2D MKDE (Table 1). Larger surface areas occurred in more uneven terrain, such as ridgelines or channels, and more in the winter range (Figure 2) than the summer range (Figure 2). Indeed, the relative percent increase in estimated surface area from 2.5D MKDE was greater for winter (14.96% to 16.82%) versus the summer range (12.09% to 12.48%; Table 1) when compared with 2D MKDE.

**Table 1:** Estimates of giant panda space use based on 2D and 2.5D MKDEs.

		Area km^2^				
Season	MKDE	99%	95%	75%	50%	n
	2D	8.159	5.030	1.940	0.821	650
All	2.5D	9.285	5.746	2.214	0.940	
	Change	13.80	14.23	14.17	14.57	
	2D	1.173	0.810	0.347	0.159	167
Winter	2.5D	1.370	0.944	0.402	0.183	
	Change	16.82	16.55	15.67	14.96	
	2D	5.592	3.405	1.352	0.572	479
Summer	2.5D	6.289	3.826	1.515	0.642	
	Change	12.48	12.38	12.09	12.25	

For area, percentages correspond to percentages of the total MDKE for which the area was estimated.

Twelve locations between the summer and winter ranges were observed and not used in the seasonal MKDEs. Percent change was calculated as 100×([2.5D MKDE area] – [2D MKDE area])/[2D MKDE area].

### California Condor

**Figure 3:**
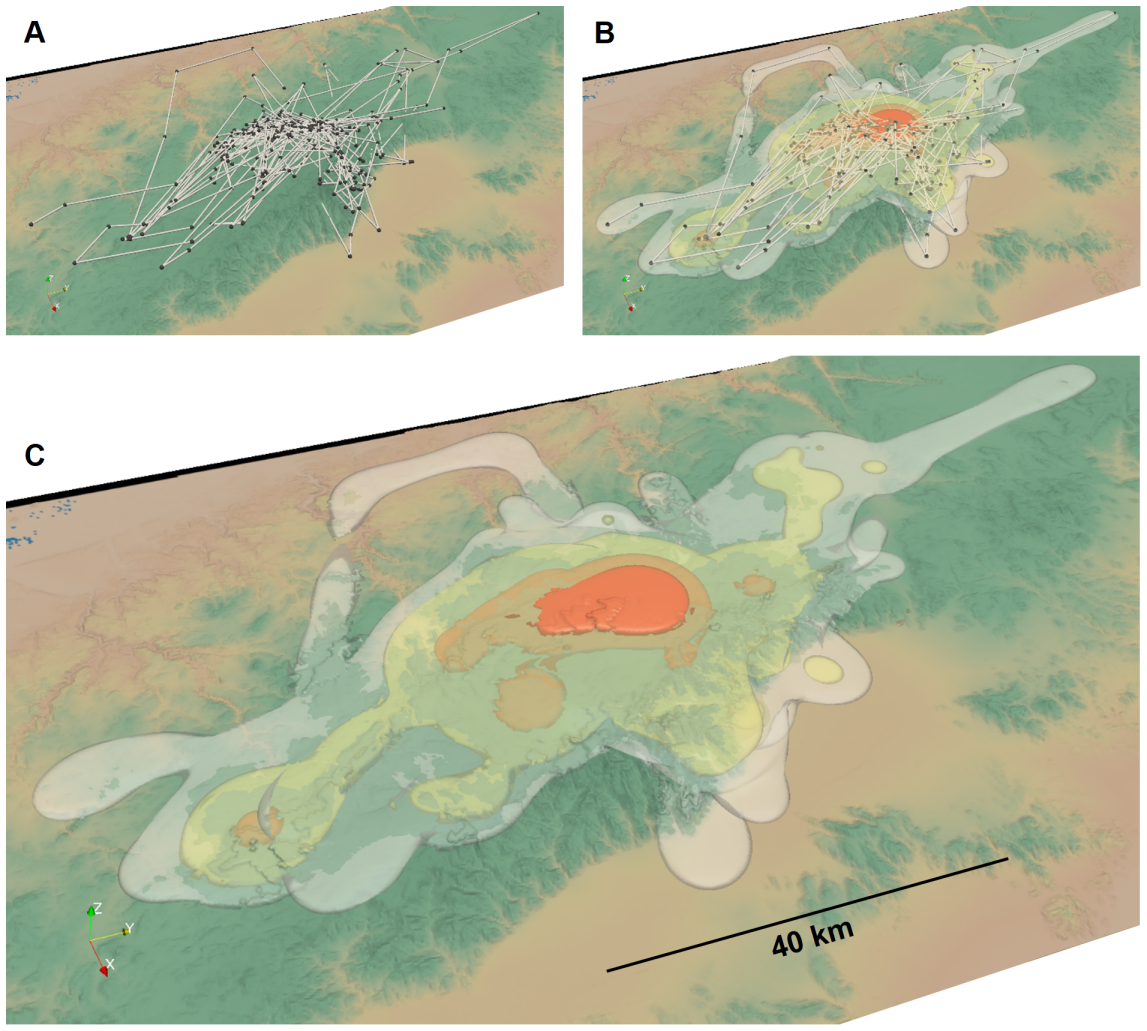
An illustration of the steps in generating a 3D MKDE for a California condor. The 3D MKDE is constructed from observed 3D locations and a digital elevation model that sets the lower bound on the MKDE. The expected location (gray points) at each unobserved time is determined by linear interpolation (white lines) between the observations (A). The 3D MKDE is then constructed by integrating a trivariate normal distribution, possibly constrained above or below in the z-dimension, over time along the interpolated movement path (B). The variance of the kernel increases as it moves further from the times of the observed locations. The contours of the final 3D MKDE is shown in C. In B and C, the 99%, 95%, and 50% 3D MKDE volumes are shown in transparent white, orange, and red, respectively.

In Figure 3, we illustrate the sequence of steps to compute a 3D MKDE. First, observed locations were filtered to ensure the move step from them occurred with the user specified time interval, that the move step most likely represented a move (rather than the differences in locations being strictly due to observation error), and that the observed *z*-coordinates fell above the digital elevation model. Of the 2,256 total observed locations, 1,253 satisfied the conditions for use in the 3D MKDE and 610 were removed because we concluded that the following location did not represent a large enough displacement to consider it a move step. Move paths were then approximated by linear interpolation (Figure 3A). Next, the variance parameters were estimated. Based on the 1253 move steps, we estimated 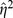  = 768993.91 m^2^/min and 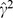  = 1612.94 m^2^/min. The kernel was then integrated over time and over each voxel to estimate the utilization probability (Figure 3B). Figure 3B shows 99%, 95%, 75%, and 50% contours along with the observed data and the interpolated move paths. Figure 3C shows an enlarged visualization of the 3D MKDE contours. Most of the condor UD (based on the 50% and 75% contours) was focused on the mountain range where it was reintroduced. Lower density use occurred in the adjacent low-lying areas.

**Figure 4:**
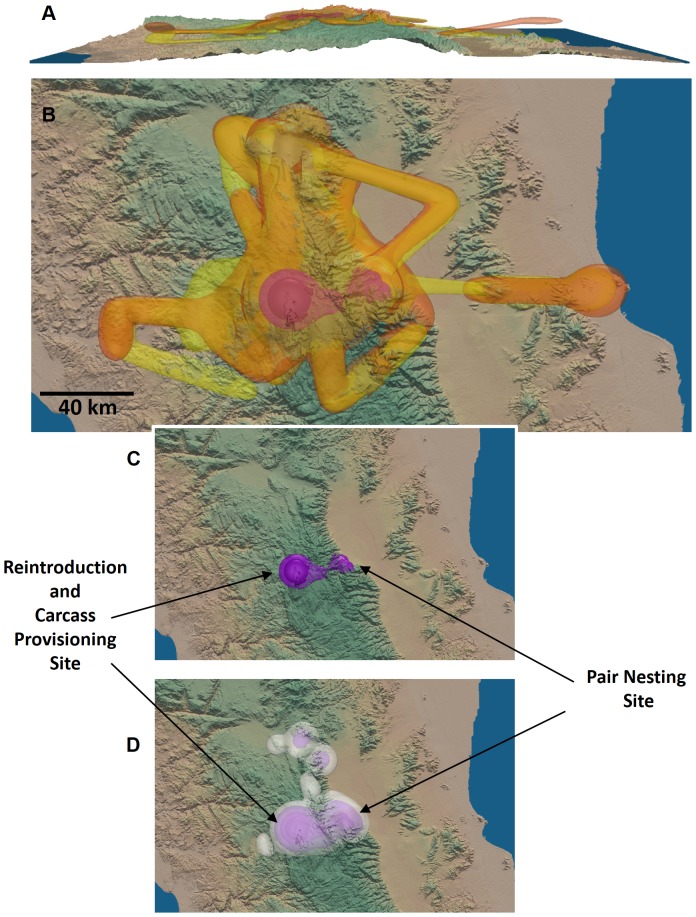
3D MKDEs for a breeding pair of California condors. First we illustrate the 99% contours for the female (orange) and male (yellow), shown as a profile view (A) and an overhead view (B). The MKDEs overlap considerably, but the male appears to spend more time at lower elevations when the pair moves into lower elevation areas. The contours for the voxels that contribute 75% and 50% of the total spatio-temporal interaction of the pair are shown in medium and deep purple (B, C). These areas correspond to the reintroduction site where condors were provisioned with carcasses following reintroduction and the nesting site for the pair. When the 99% (white) and 95% (light purple) contours shown, several other areas are included which may also be of ecological interest.

Next, we considered the breeding pair of condors. Of the 1,132 total observed locations for the female condor, 501 satisfied the conditions for use in the 3D MKDE. Based on the 501 move steps, we estimated 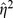  = 465862.25 m^2^/min and 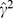 = 1212.08 m^2^/min. Of the 1132 total observed locations for the male condor, 550 satisfied the conditions for use in the 3D MKDE. Based on the 550 move steps, we estimated 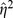  = 702010.65 m^2^/min and 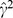  = 1562.60 m^2^/min. Comparing the side-view of the 3D MKDEs for each condor shows that the female tended to be at lower altitudes when the pair moved into the lower-elevation areas to the east and west of the mountain range where they were most active (Figure 4A). However, there was a substantial overlap of the 3D MKDEs (Figure 4B). We also applied the MKDE-based estimate of spatio-temporal similarity to the data for the condor pair. Contours around the minimum volume encompassing 75% of the voxels with the highest spatio-temporal association identified two areas. The first is the nesting site for the breeding pair (Figure 4C). The second was the release site and the area where reintroduced condors were provisioned with food. This area was also identified by the 50% contour. Without this *a priori* knowledge, we could have identified these areas using the MKDE-based spatio-temporal interaction method. When we consider the 95% and 99% interaction contours (Figure 4D), other areas where the pair interacts most often were identified, and may warrant further investigation. The global measure of similarity between the two condors was 0.6013, which indicates considerable spatio-temporal overlap in their utilization distributions.

**Figure 5:**
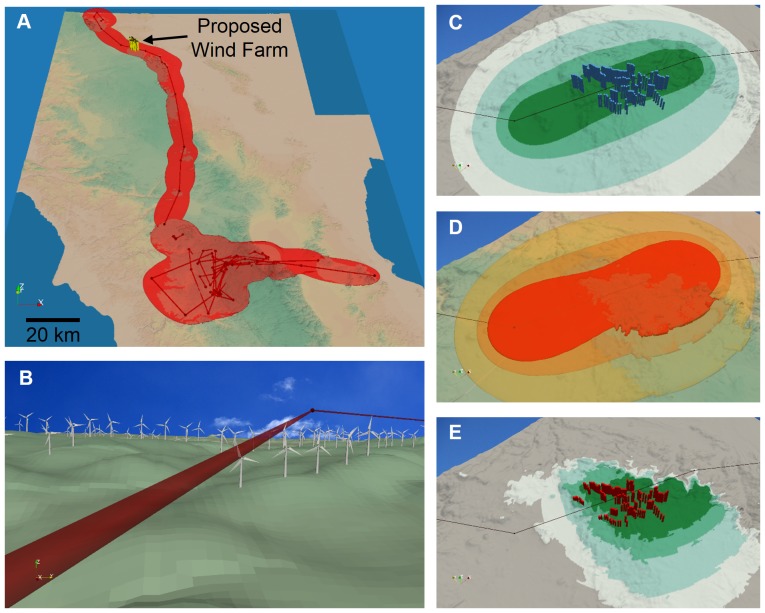
3D MKDEs may help better identify threats to species. In 2007, a subadult female condor made an exploratory movement through a proposed wind energy development (A). The proposed wind turbine locations are shown in yellow, and the 99% contour for the condor is shown in red. When approximating the condor's move path by linearly interpolating between observed locations (red lines, A–E), the path passes through the proposed locations of the wind turbines (B). The 3D models of 120 wind turbines are shown (B) in their proposed locations and size (Vestas V112-3.3 turbines with a 84 meter hub height and a 56 meter rotor radius). Using 2D and 3D MKDEs, we estimated the probability that the condor would have passed through cells (54 meters square, C) and voxels (54 meters cubed, D–E) intersecting each turbine. The 99%, 95%,75%,and 50% contours are shown for the 2D MKDE, and the height of blue 3D bars at each turbine location indicate the probability that the condor passed through cells intersected by the turbines (C). The 95%, 75%,and 50% contour volumes are shown for the 3D MKDE (D, the 99% contour was omitted because it covered most of the topography). For comparison to (C), the 99%, 95%,75%,and 50% contours for the three levels of voxels closest to the ground (the approximate height of the turbines) are shown, and red 3D bars at each turbine location indicate the probability that the condor passed through voxels intersected by the turbines (E). The height of the bars in (C) and (E) are on the same relative scale. In general, the probabilities based on the 3D MKDE are lower and more closely related to the observed altitudes of the condor, the possible altitudes it may be at when it is not observed, and the terrain.

Next, we consider the interaction between historical movements of a subadult female condor and placement of a proposed wind farm. Of the 333 total observed locations, 134 satisfied the conditions for use in the 3D MKDE and 74 were removed because we concluded that the following location did not represent a large enough displacement to consider it a move step. Based on the 134 move steps, we estimated 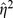  = 833361.81 m^2^/min and 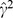  = 2538.53 m^2^/min. The 3D MKDE in relation to the proposed wind farm project area is shown in Figure 5A. Linearly interpolating the move path between observed locations suggests that the condor would have passed though the wind farm and that there would have been a risk of collision with the turbines.

We estimated the 2D and 3D MKDEs using the single move step through site (Figure 5B). Variance parameters for this MKDE were estimated from 23 locations acquired during the exploratory movement bout, yielding estimates of 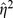  = 1004416.46 m^2^/min and 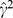  = 1462.03 m^2^/min. From these MKDEs, we extracted the probabilities associated with each wind turbine to assess the risk the wind farm would have posed to the condor. Based on the 2D MKDE, the turbine encounter probabilities ranged from 5.180e-06 to 8.861e-06, with a mean of 7.988e-06 and standard deviation of 7.141e-07 (Figure 5C). For the 3D MKDE (Figure 5D), turbine encounter probabilities ranged from 1.946e-06 to 6.777e-06, with a mean of 4.659e-06 and standard deviation of 1.106e-06 (figure 5E). The risk ratios, defined as the 2D encounter probability divided by the 3D encounter probability, ranged from 1.182 to 4.276, with a mean of 1.845 and a standard deviation of 0.633. Thus, while the probabilities are correlated (r = 0.2314, p-value  = 0.01099), the 2D probabilities are higher. Furthermore, the probabilities from the 3D MKDE are related to the topography, the observed altitudes of the condor, and the possible altitudes that the condor could have been at times it was not observed, whereas those from the 2D MKDE are not (Figures 5C and 5E).

**Figure 6:**
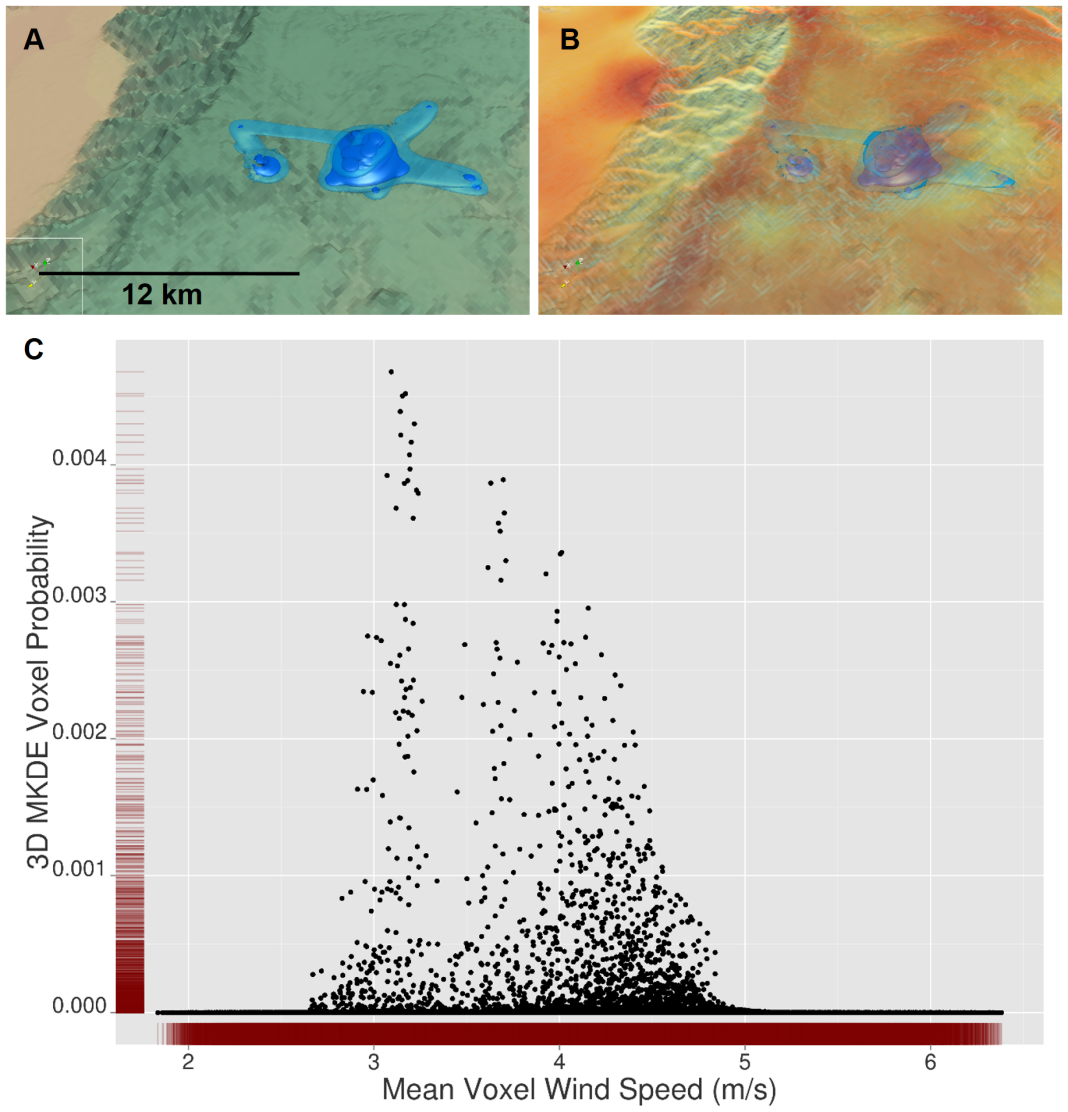
3D MKDE Voxel Probabilities and 3D Covariates. Like 2D utilization distributions (UDs), 3D UDs can be related to 2D and 3D habitat covariates. In A, we show the 99% and 95% contour volumes for an adult female condor during the month of November, 2009. In B, we show a volume rendering for predicted mean wind speed (meters/second) for November 2009 in voxels 250 m by 250 m by 10 m (x, y, z, respectively) for 0 to 150 m above the ground. Wind speed increases as the color transitions from pale yellow to red. In C, we relate the probability of the condor being in a voxel to the predicted voxel wind speed for each voxel within 150 meters of the Earth's surface. Rug plots (in red) show the marginal distribution of each variable.

In the final condor example, we relate MKDE probabilities to predicted wind speed using 292 of 433 total locations that satisfied the conditions for use in the 3D MKDE, where 118 move steps were removed because we concluded that they were non-movements. Based on the 292 move steps, we estimated 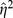  = 23801.01 m^2^/min and 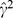  = 84.72 m^2^/min. The resulting 3D MKDE is illustrated in Figure 6A, and is shown with the wind speed predictions in Figure 6B. When relating the voxel 3D MKDE probabilities and predicted wind speed, a scatter plot suggests that higher voxel probabilities may be associated with intermediate predicted wind speeds (Figure 6C).

### Dugong

**Figure 7:**
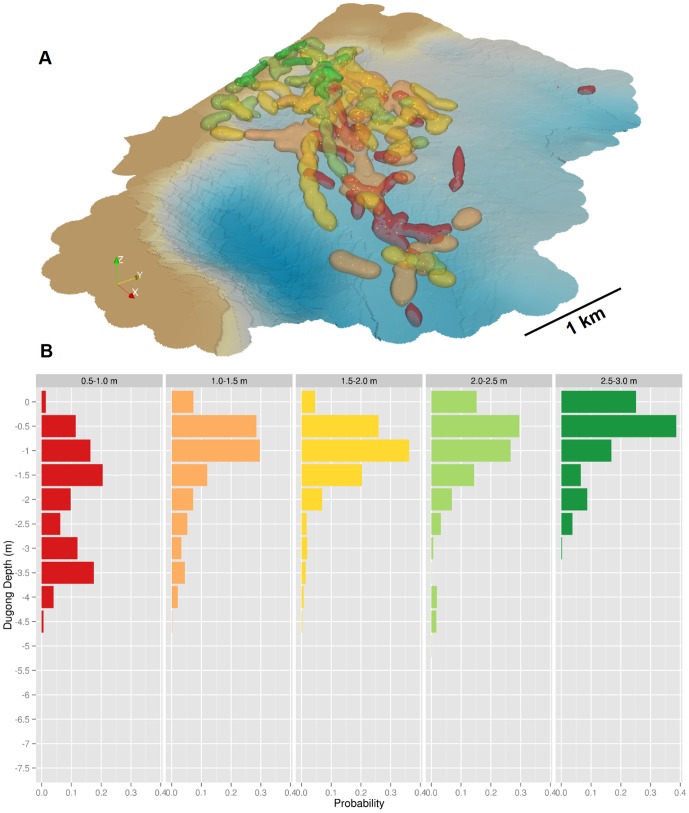
Examples of a 3D MKDE for a dugong in a marine environment. Dugong 3D MKDE density is visualized in relation to bathymetry (A). The 99% contour volumes for 3D MKDEs based on locations when tidal heights ranged from 0.5–1.0 (red), 1.0–1.5 (orange), 1.5–2.0 (yellow), 2.0–2.5 (light green), and 2.5–3.0 (green) meters are shown. Based on the 3D MKDEs for each tidal height category, we computed the probability that the dugong would have been at different water depths, grouped in 0.5 meter bins (B). The value on the y-axis is the upper depth value for each 0.5 meter bin (i.e. 0 indicates 0.0–0.5 m depth).

Figure 7A illustrates 3D MKDEs for the dugong at tidal ranges of 0.5–1.0, 1.0–1.5, 1.5–2.0, 2.0–2.5, and 2.5–3.0 meters. Variance estimates and estimated volumes within several percentages of the UD are given in Table 2. It is clear from Figure 7 that for higher tides, the dugong spent more time closer to shore and in shallower water. As the tide became lower, the dugong tended to move further away from shore and use greater depths (after adjusting for tidal height). This pattern is shown more clearly in Figure 7B, which uses bar plots to illustrate the probability that the dugong will be present in water depths divided into 0.5-meter bins for each tidal height range. The probabilities were obtained by summing the voxel probabilities within each water depth bin. For tidal height ranges of 0.5–1.0, 1.0–1.5, 1.5–2.0, 2.0–2.5, and 2.5–3.0 meters, the probability that the dugong was in the 1.5-meter detection zone was 0.292, 0.655, 0.667, 0.715, 0.806, respectively and the 2.5-meter detection zone was 0.595, 0.847, 0.939, 0.928, 0.960, respectively. This suggests, counterintuitively, that this particular dugong would have had a higher detection probability during high tides because it spent more time in shallow water near shore. In contrast, during lower tides this dugong spent more time foraging in deeper water, which would have decreased detectability. For comparison, Pollock et al. [58] reported constant, population-level probabilities of 0.47 and 0.67 for the 1.5-meter and 2.5-meter detection zones, respectively.

**Table 2:** Dugong Results by Tidal Height.

	Variance Estimates	Volume (m^3^)						
Tidal Height (m)	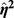	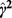	99%	95%	90%	75%	50%	n
0.5–1.0	1.197E+02	6.329E-05	1.185E+06	7.296E+05	5.370E+05	2.836E+05	1.130E+05	100
1.0–1.5	3.212E+02	5.894E-05	3.200E+06	1.993E+06	1.475E+06	7.838E+05	2.923E+05	145
1.5–2.0	2.942E+02	2.197E-04	3.422E+06	2.173E+06	1.598E+06	8.141E+05	2.945E+05	145
2.0–2.5	2.043E+02	2.527E-03	1.772E+06	1.074E+06	7.849E+05	4.144E+05	1.623E+05	113
2.5–3.0	8.694E+01	2.182E-04	4.305E+05	2.757E+05	2.051E+05	1.084E+05	4.100E+04	45

Variance parameters are in units of m^2^/minute.

Volumes correspond to volumes within each 3D MDKE contour.

n indicates the number of locations used in the 3D MKDE.

## Discussion

Our 3D home range estimators and visualizations offer considerable theoretical benefits over traditional 2D techniques. First, we were able to visually explore the 3D MKDE volumes of each example species to more intuitively understand how they spatially related to the environmental covariates and bounding layers within their ranges, such as bathymetry or topography. Second, by integrating the vertical component of animal movements into home range estimates, 3D estimators are more accurate and biologically realistic than their 2D counterparts. For example, the giant panda 2D MKDE had a much lower estimate of home range surface area than the 2.5D MKDE that took terrain into account. Home range size is positively associated with extinction risk [5], [9], suggesting that extinction risk may be systematically underestimated among species occupying rugged terrain. Similarly, life-history comparisons across species [59], [60] will be inaccurate unless biases in home range are corrected for species inhabiting mountains and other rugged terrain. Finally, 3D MKDEs can be used to examine how individuals spatially interact in 3D (Figure 4) and these interactions can be incorporated into studies of resource and niche partitioning among ecologically-similar species [11].

The 2.5D MKDEs allow more realistic inferences to be drawn regarding giant panda habitat use. Pandas are known to make use of seasonal food resources that vary with elevation [37] and to select microhabitats associated with topographic features that support intraspecific chemical signaling [61]. Thus, incorporating the vertical dimension in the analysis can improve understanding of habitat use patterns that vary with topography. In addition, 2.5D MKDEs provide greater insight into seasonal variations in panda migration paths and the degree of spatiotemporal overlap among conspecifics. Finally, bamboo forage and the old growth trees that pandas preferentially select as breeding dens are increasingly under pressure from human disturbances, such as logging and climate change [62]. Hence, strategies for panda conservation should incorporate understanding of important topographic and elevational determinants of habitat requirements.

Dugongs exhibit strong spatial association with seagrass patches of relatively elevated nutritional quality and quantity [54]. Thus, the improved accuracy of our 3D MKDE over traditional 2D estimators will enable the space use of dugongs to be more closely matched to the attributes of the seagrass pastures they inhabit. Greater understanding of dugong interaction with food resources will in turn enhance conservation management efforts to identify, delineate, and protect important dugong seagrass habitat. Furthermore, 3D models enable the proportion of dugong home ranges within shallow and deep water zones to be defined, providing better predictions of the risks posed to dugongs by drowning in bottom-set fishing nets or injury by boat strikes. Finally, 3D MKDE may increase the accuracy of aerial surveys of dugong populations by incorporating volumetric home ranges into probability estimates of detectability across the zones of water depths dugongs inhabit (Figure 7, [58]). In future work, we will use 2D and 3D MKDEs to further explore how space use depends on environmental covariates varying at short temporal scales, such as tidal height.

Volumetric MKDE home ranges enable condor spatial behaviors to be matched with the environmental covariates, such as wind speed or other climatic conditions that modify flight behaviors, in 3D (Figure 6). In addition, the risk of injury to condors colliding with wind turbines that increasingly coincide within their expanding ranges could be better predicted, as volumetric condor ranges can be intersected with existing or planned wind farms and the degree of spatial overlap quantified to provide more accurate estimates of collision probabilities (Figure 7). When applying this approach to support real-world applications, great care should be taken to compute the collision probability over a volume that closely corresponds to that occupied by the wind turbines. Use of 3D MKDE suggests a lower probability of risk of turbine collision compared to the 2D MKDE, and provides more specific guidance to mitigate bird injuries and mortalities because it accounts for condor elevation and topography.

The benefits of 3D home range estimation can extend to other circumstances where there is a vertical component to animal movement. For example, understanding the role of vertical stratification in resource partitioning in arboreal species is an especially promising future application of this technique, and may help explain the high biodiversity found in tropical forests [63]. The 3D MKDE also could be extended to model the home ranges of fossorial species within a bounding, underground matrix, as well as to incorporate temporally changing constraints on the space available to an animal in the *z*-dimension. The 3D MKDE could also be used to calculate resource selection functions in 3D to determine the frequency at which habitats are used by animals disproportionately to their availability across a landscape [64].

3D MKDEs can enhance the ecological basis of conservation management strategies for mitigating anthropogenic impacts on threatened populations of vagile wildlife. For example, analyzing the 3D movements of avifauna in relation to the spatio-temporal distribution of aircraft flight paths, power lines, or buildings will provide more accurate estimates of collision risk than 2D models. Improved understanding of the 3D spatial behaviors of the many aquatic animals currently being tracked with biologgers, such as marine turtles, would help managers to minimize their incidental capture by fisheries. 3D MKDEs could also be incorporated into predictive models of wildlife exposure to soil, air and water borne contaminants, and used in simulations of the effects of changing water temperatures, currents or acidification on threatened populations. Although no home range estimator is uniformly superior, our 3D MKDE is a significant step towards Burt's original 1943 concept of a home range and timely leap out of 2D “Flatland”. Wildlife biologists and conservation managers may now analyze their biotelemetry data sets in all three spatial dimensions to visualize and estimate animal space use that can be more realistic, accurate, and informative than those calculated using 2D methods.
