# Correction: Movement-Based Estimation and Visualization of Space Use in 3D for Wildlife Ecology and Conservation

**DOI:** 10.1371/journal.pone.0109065

**Published:** 2014-09-23

**Authors:** The PLOS ONE Staff

The words red and blue are swapped in the legend of Figure 2. The complete, correct Figure 2 legend is:

**Figure 2:**
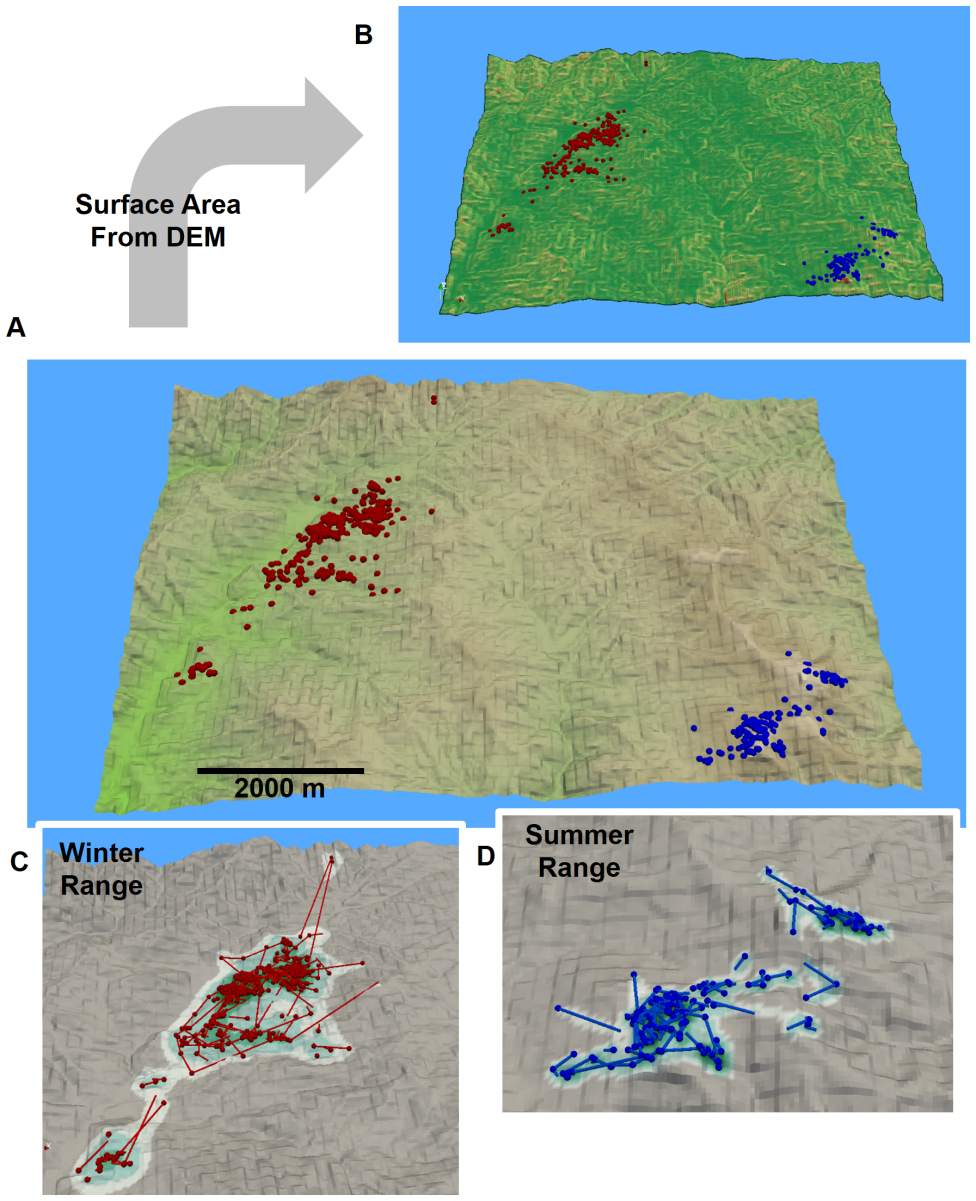
An example of a 2.5D MKDE for a giant panda in rugged terrain. In A, giant panda GPS locations in its summer (blue points) and winter (red points) ranges are shown in relation to a digital elevation model (DEM). Using the DEM, the surface area of each raster cell is calculated (B). The surface area increases as the color gradient changes from green to red. In C, the observed summer range locations and interpolated move paths (blue points and lines) are shown against 2D MKDE contours draped over the DEM. In D, the observed winter range locations and interpolated move paths (red points and lines) are shown against 2D MKDE contours draped over the DEM. 2D MKDE 99%, 95%, 75%, 50% contours are shown with colors ranging from light to dark green.
